# Co1 DNA supports conspecificity of *Geomyphilus pierai* and *G. barrerai* (Coleoptera, Scarabaeidae, Aphodiinae) and is a good marker for their phylogeographic investigation in Mexican mountains

**DOI:** 10.3897/zookeys.512.9646

**Published:** 2015-07-06

**Authors:** Alfonsina Arriaga-Jiménez, Lise Roy

**Affiliations:** 1 UMR 5175 CEFE, CNRS - Univ Montpellier - Université P. Valéry - EPHE, Route de Mende 34199 Cedex5. France; 2 Instituto de Ecología, A.C. Carretera antigua a Coatepec 351, El Haya, Xalapa 91070, Veracruz, Mexico

**Keywords:** Dung beetles, pocket gophers, phylogeography, Co1, ITS, Mexico

## Abstract

Members of *Geomyphilus* are associated with rodent burrows, such as pocket gophers and prairie dogs. In Mexico, they are found in the mountains of the Mexican Volcanic Belt and in Sierra Madre Oriental. Our study aims to initiate the exploration of the dispersal modes of *Geomyphilus pierai* and *Geomyphilus barrerai* from burrows of pocket gophers. In order to estimate the dispersal scale of the beetles, the utility of mitochondrial and nuclear molecular markers for studying the phylogeographic structure of this complex of species (*Geomyphilus pierai* and *Geomyphilus barrerai*) was tested from 49 beetle individuals. High intraspecific and intra-mountain nucleotidic diversity was captured from this sample using Co1 mitochondrial sequences, whilst the ITS2 nuclear ribosomal sequence did not allow observing informative variation. Mitochondrial phylogenetic analysis revealed that the specific delineation between the two species under study was doubtful. In this preliminary study, Co1 was shown to be a good marker for elucidating dispersal routes of the burrowing rodent-associated beetles.

## Introduction

The subfamily Aphodiinae is one of the most diversified groups within the Scarabaeoidea (classification *sensu* Dellacasa et al. 2001) showing a worldwide distribution (Dellacasa 1987, 1988a, b, 1991, 1995) and having a remarkably high generic and specific diversity. The Aphodiinae are the predominant group in the communities of the dung beetles in the cold temperate areas of the Palearctic region (Hanski 1991) and also in the Nearctic region (Lobo 2000).

Members of *Geomyphilus* are endemic of the Nearctic region and are associated with rodent burrows. Common hosts include pocket gophers (Geomyidae) and prairie dogs (Sciuridae: *Cynomis*), but some data indicate that rarer Aphodiinae species may be found in association with kangaroo rats (Heteromyidae: *Dipodomys*), voles (Cricetidae: *Microtus*), or other rodents (Gordon and Skelley 2007). In Mexico, members of *Geomyphilus* are found in the mountains of the Mexican Volcanic Belt (MVB) and in Sierra Madre Oriental, including the two presumed sibling species: *Geomyphilus pierai* (Lobo & Deloya, 1995) and *Geomyphilus barrerai* (Islas, 1955).

While the habits and distribution patterns of their associated rodents are rather well-known (Demastes et al. 2002; Hafner et al. 2005; Fernández et al. 2014), these beetles’ dispersal modes were unknown to date. Most of the southern pocket gophers, such as *Cratogeomys merriami* and *Thomomys umbrinus*, live at high elevations in the Mexican mountains. Their burrowing activity results in a net of galleries under the ground in many habitats of these mountains. The two species we are focusing on (*Geomyphilus pierai* and *Geomyphilus barrerai*) are known to live in close association with pocket gophers, with the beetles recurrently found nesting in and feeding on the rodent feces (Lobo and Halffter 1994, Deloya and Lobo 1995, Arriaga-Jiménez pers. obs.). These nidicolous species were up to now only found underground, inside the rodent nests, latrines or galleries, with very few exceptions. *Geomyphilus* beetles possess a priori functional wings, which could allow a long distance spread by flying. However, the fact that the beetles live in a close association with rodents and that they were mainly found underground suggests that these scarabs may present mainly a short distance spread by going through the gophers’ galleries. Alternatively, the rarity of the collecting outside the burrows might be a result of undersampling due to the short flight period of the beetles.

We undertook a preliminary molecular study to investigate *Geomyphilus* dispersal modes and clarify whether the preferred route is underground or aerial. Measuring the amount of gene flow between the populations in different mountains could help understanding the spatial scale of the *Geomyphilus* dispersal. Indeed, one may presume that if the species are mainly moving within the rodent galleries, the gene flow between the populations in different mountains should be less than if they fly outside.

To initiate the exploration of the dispersal routes of coprophagous scarabaeids narrowly associated to rodents, the development of molecular genotyping is needed, allowing to characterize the population genetic structure at different spatial scales within at least one species living in rodent burrows. Candidate molecular markers should provide genetic variation detectable at spatial scales appropriate to assess aerial dispersal. We assumed that the aerial dispersal might allow individuals to quickly fly from one mountain to another, whilst the underground dispersal most likely would limit the beetle circulation to a single mountain or at most to the interconnected mountains. Therefore, we decided to work on the individuals sampled from the nests located in the different mountains of MVB.

Moreover, several species of the genus *Geomyphilus* can be found together in the same gopher nest (Deloya and Lobo 1995, Gordon and Skelley 2007, Skelley et al. 2007, Arriaga-Jiménez, pers. obs.) and the two above cited species were recurrently found together in rodent nests by AAJ (during her PhD fieldwork). Because of such a co-occurrence and due to the rather difficult morphological discrimination between them, the interspecific molecular delineation was needed in first.

**Figure 1.**
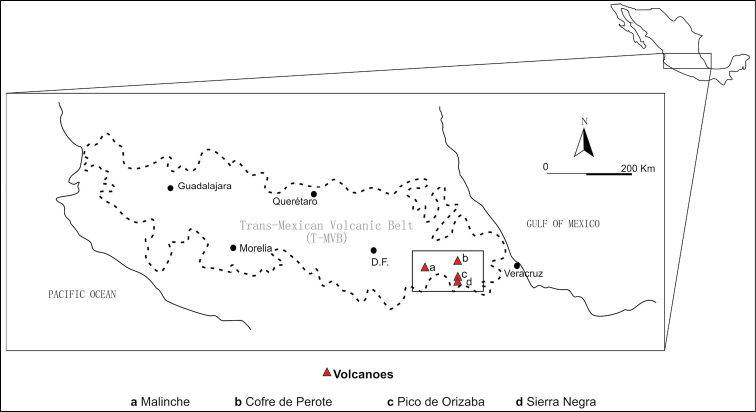
Sampled mountains are in the Eastern part of the Trans-Mexican Volcanic Belt: Malinche, Cofre de Perote, Pico de Orizaba and Sierra Negra.

Here we present the results from a preliminary molecular investigation of *Geomyphilus pierai* and *Geomyphilus barrerai* aiming at (1) assessing interspecific molecular divergence and (2) testing molecular markers to allow phylogeographic studies to be performed in these species. We sequenced one mitochondrial and one nuclear DNA regions from individual beetles sampled from the burrows of pocket gophers (*Cratogeomys merriami* and *Thomomys umbrinus*) in four mountains of the MVB. The mountains under study were: La Malinche, Cofre de Perote, Pico de Orizaba and Sierra Negra (Fig. 1). The first one is separated from the others by the High Plateau, while the other three have some connectivity, especially Pico de Orizaba and Sierra Negra which are located next to each other.

## Methods

**Figure 2.**
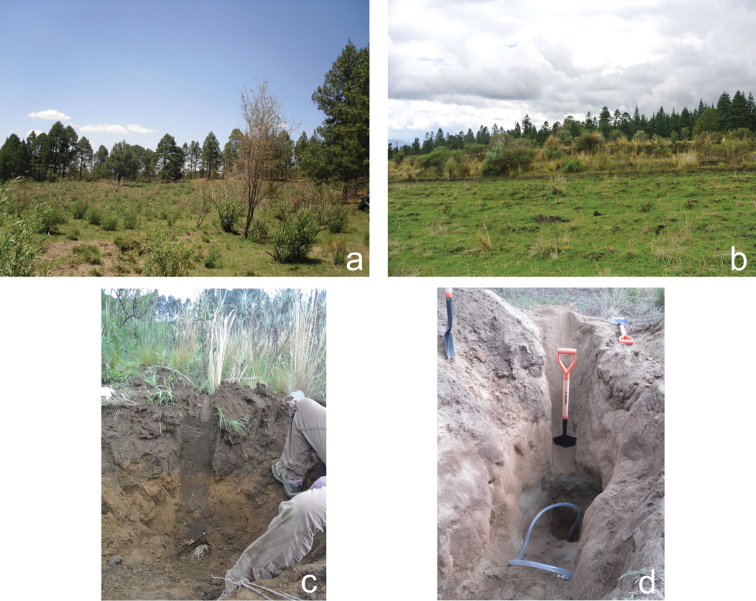
Example of the biotopes where the pocket gophers’ burrows were dug: **a** scrubland in Malinche **b** grassland in Sierra Negra **c** pocket gopher nest at 50 cm depth **d** pocket gopher nest at more than 1.6 m depth.

To collect *Geomyphilus* beetles, the gopher nests were excavated (only those that seemed recently used). The galleries were followed up to find the nests and latrines where the dung beetles might be found. This sampling technique is very effort-consuming because the nests can be located very deep and they are not always found. Once the nests and latrines were reached, the organic matter was removed as well as the soil within 20 cm from it, and then all the soil was carefully inspected for scarabs. The depth of the nest and latrines varies depending on the type of soil, slope and surrounding vegetation. They were found from 30 cm to 1.5 m deep in different biotopes (scrublands and grasslands) in the studied mountains (Fig. 2). All the individuals were immediately transferred to 90% ethanol and conserved for further morphological identification and molecular analyses. All specimens examined were identified by Giovanni and Marco Dellacasa.

The DNA was extracted from ethanol-preserved specimens. Either of two different techniques were used, both designed to obtain molecular information while keeping voucher cuticle of each individual. For the first one, after complete evaporation of ethanol, the cuticle of each specimen was cut at two points on the prothoracic-mesothoracic joint soft integument in order to facilitate the lysis of internal tissues. This was done with a sterile syringe needle on a glass slide before insertion of individual in a vial for proteinase K digestion. For the second technique, one leg was removed from the beetle, then put in an Eppendorf vial and crushed with a pestle. For both techniques, the entire cut individual or the crushed leg were immersed in 100 ml Buffer ATL + 15 ml Proteinase K and incubated for 19–30 h at 70 °C, before proceeding to the DNA purification using Qiagen’s DNeasy Blood & Tissue Kit following the manufacturer’s protocol. The specimens’ cuticles (tech. 1) were conserved in glycerol and the body remaining (tech. 2) in ethanol as vouchers.

Checking congruence between phylogenetic topologies from genes with different transmission patterns such as mt DNA (maternal transmission) and nuclear DNA (biparental transmission) is useful to confirm cryptic species (see Donelly et al. 2013). For this purpose, Co1 and IT2 appeared to be good candidates, since they were shown to be variable both at the inter- and intraspecific levels in some scarab beetle genera (Wirta 2009). Two fragments from one mitochondrial protein-coding gene (Co1) and one nuclear ribosomal internal transcribed spacer (ITS2) gene were amplified. Primer pairs used for amplification of Co1 were LCO_1490 and HCO_2198 (Folmer et al. 1994) or LEP-F1 and LEP-R1 (Hebert et al. 2004); and for the nuclear region ITS2 we used ITS2f (Navajas et al. 1998) and RhITSR (De Rojas et al. 2002).

Polymerase chain reaction (PCR) was performed using the Qiagen’s Taq PCR Core kit. The following reagent concentrations were used for a final volume of 25 µL per tube: 1×PCR buffer, 0.036U/µl Qiagen Taq Polymerase, 300 µM dNTPs and 0.6 µM of each Co1 primer or 0.43 µM of each ITS2 primer, and 2.5 mM MgCl_2_ for CO1 or 3.0 mM MgCl_2_ for ITS2. For the Co1, thermal cycling parameters comprised an initial denaturation step of 10 minutes at 94 °C, followed by 5 cycles of 94 °C for 40 s, 49 °C for 60 s and 62 °C for 60 s, and 35 cycles of 94 °C for 40 s, 52 °C for 60 s and 62 °C for 60 s, with a final elongation at 62 °C for 10 min. For the ITS region, thermal cycling parameters comprised an initial denaturation of 10 minutes at 94 °C, followed by 5 cycles of 94 °C for 40 s, 56 °C for 60 s and 72 °C for 60 s, and 35 cycles of 94 °C for 52 s, 52 °C for 60 s and 72 °C for 60 s, with a final elongation at 72 °C for 10 min. Purification and sequencing was realized by Genoscreen (France, Lille) using a 96-capillary sequencer ABI3730XL.

Obtained DNA sequences were aligned using Muscle (Edgar 2004) in Seaview4.1 (Galtier et al. 1996). The absence of any stop codon was first checked in the Co1 sequence alignment. Maximum likelihood phylogenetic analyses were performed using PhyML (Guindon and Gascuel 2003) and nucleotidic diversities were compared between samples and between taxa using DNAsp 5.10.01 (Rozas and Rozas 1995; Librado and Rozas 2009). A median-joining network (Bandelt et al. 1999) of DNA fragments was constructed using NETWORK v4.5.1.6 (fluxus-engineering.com) to visualize the frequency and geographical distribution of Co1 haplotypes.

## Results and discussion

**Figure 3.**
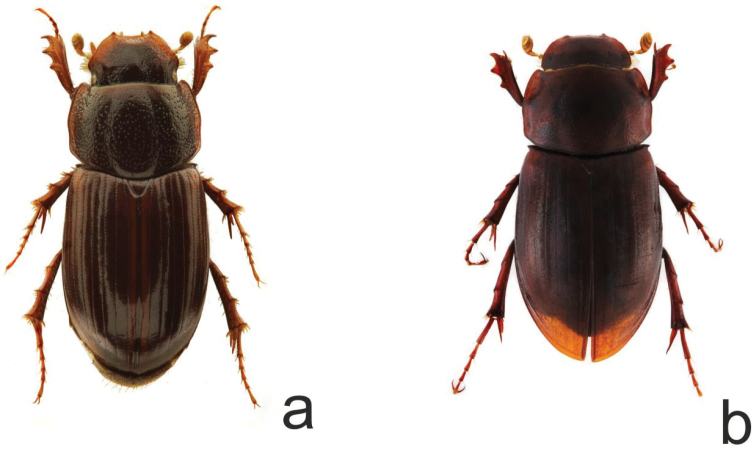
Habitus of adults **a** *Geomyphilus pierai* **b** *Geomyphilus barrerai*.

**Figure 4.**
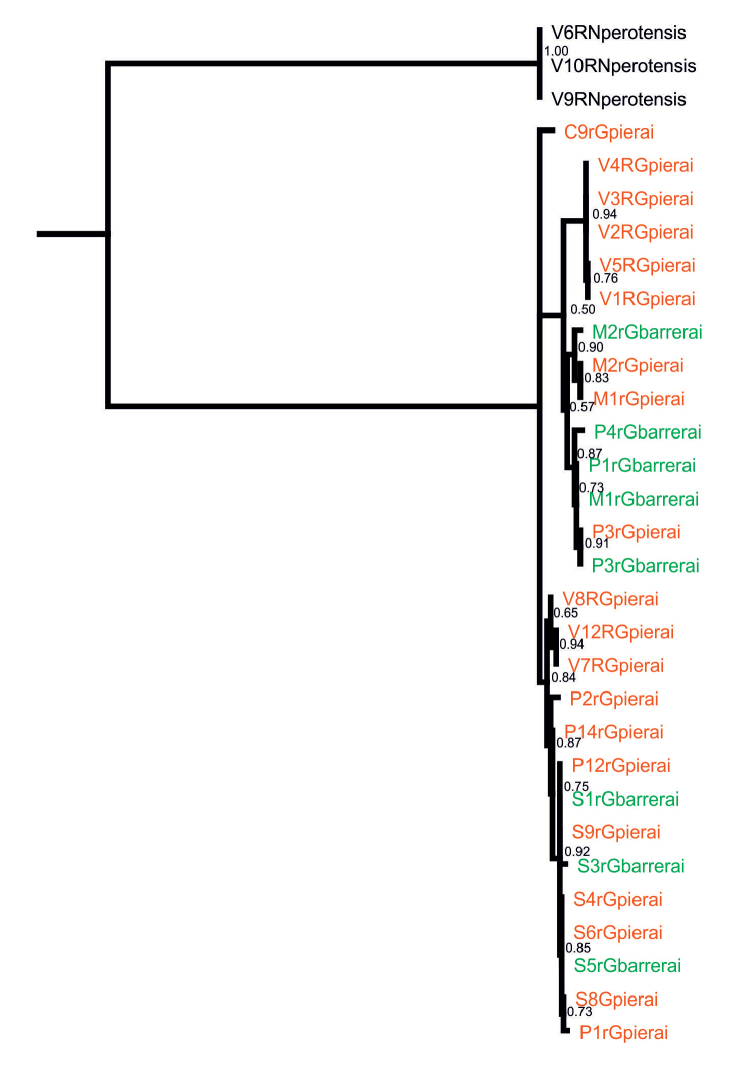
Label color indicates the morphological taxonomic assignation of each individual: red, *Geomyphilus pierai*, green, *Geomyphilus barrerai*, black, outgroup. Numbers at nodes are bootstrap values (500 rep.; only values ≥0.5 are provided).

In order to estimate the utility of the two DNA markers to assess the phylogeographic structure of this complex of species (*Geomyphilus pierai* and *Geomyphilus barrerai*; Fig. 3), a 700-bp Co1 fragment was obtained from 28 individuals and a ca. 830-bp ITS2 fragment from 47 individuals collected in 21 rodent nests distributed across the four different mountains of the MVB (total 49 sequenced individuals, see Online supplementary materials). Intraspecific and intra-mountain diversity was captured from this sample using mitochondrial sequences, not nuclear sequences (Table 1). Indeed, 17 Co1 haplotypes were recorded among individuals identified as belonging to either *Geomyphilus pierai* or *Geomyphilus barrerai*, with 46 polymorphic sites, a 4.1 mean pairwise percent of nucleotidic differences and a 0,963 Hd value (haplotype diversity). In contrast, 4 ITS2 haplotypes were recorded, with only 3 polymorphic sites (Figure 4). Not only differences were very scarce among the nuclear DNA fragments, but also a single haplotype was largely dominating, contrarily to what was observed in some other scarab species (e.g. Wirta 2009). No clear association between any mitochondrial lineages was noticed and the largely dominant haplotype was also found in the three outgroup individuals (*Neotrichonotulus perotensis*). As a result, we considered this marker inappropriate to investigate phylogeographics in *Geomyphilus* spp. and only performed subsequent analyses on the Co1 dataset.

**Table 1.** Nucleotide diversity: (Pi, Dxy and Da) between *Geomyphilus pierai* and *Geomyphilus barrerai*.

**Pi**				
	Cofre-Vigas	Pico Orizaba	Sierra Negra	Malinche
Cofre Perote	-			
Pico Orizaba	0.02305	-		
Sierra Negra	0.01918	0.01622	-	
Malinche	0.02143	0.02083	0.0193	-
				
Dxy				
	Cofre-Vigas	Pico Orizaba	Sierra Negra	Malinche
Cofre Perote	-			
Pico Orizaba	0.02605	-		
Sierra Negra	0.025	0.01898	-	
Malinche	0.02615	0.02286	0.034	-
				
Da				
	Cofre-Vigas	Pico Orizaba	Sierra Negra	Malinche
Cofre Perote	-			
Pico Orizaba	0.00623	-		
Sierra Negra	0.01439	0.00725	-	
Malinche	0.01206	0.00765	0.02799	-

Species boundaries between *Geomyphilus barrerai* and *Geomyphilus pierai* were not confirmed either by mitochondrial or nuclear data. When considering concordance between morphological taxonomic assignation and molecular information, not only none of the 46 Co1 polymorphic sites and none of the 4 ITS2 polymorphic sites did segregate accordingly, but also, the distribution of the two taxa across the Co1 topology was dispersed and intermingled, without any clade grouping together all individuals of any of them (Fig. 4). Although some other cryptic species (different than either *Geomyphilus barrerai* or *Geomyphilus pierai*) could eventually be revealed by using a more rapidly-evolving nuclear gene, the current specific delineation is most likely unable to match any actual sexual isolation between these current taxa. If two or more other cryptic lineages were confirmed by some other nuclear analyses, a wide morphological screening involving genotyped individuals from diverse origins would be required to improve specific description and allow determining whether any characters are both discriminant between these entities and stable within each of them. This is strengthened by the fact that higher nucleotidic diversity was found in Co1 sequences between mountains than between taxa (see Table 2). All that strongly suggests that these two taxa represent either a single biological species or a differently structured species complex. As a result, *Geomyphilus pierai* should be considered for synonymization with *Geomyphilus barrerai* following the ICZN principle of priority.

**Table 2.** Nucleotide diversity between *Geomyphilus pierai*, *Geomyphilus barrerai* and *Neotrichonotulus perotensis*.

	Gb	Gp	Gb vs Gp	Gp vs Np	Gb vs Np
n	8	20	28 (20 and 8)	20/3	8/3
k	13.8570	14.5630	15.0030	30.6800	43.6000
Pi	0.0208	0.0219	0.0225	0.0461	0.0654
Dxy			0.0236	0.1250	0.1256
Da			0.0023	0.1141	0.1152

**Figure 5.**
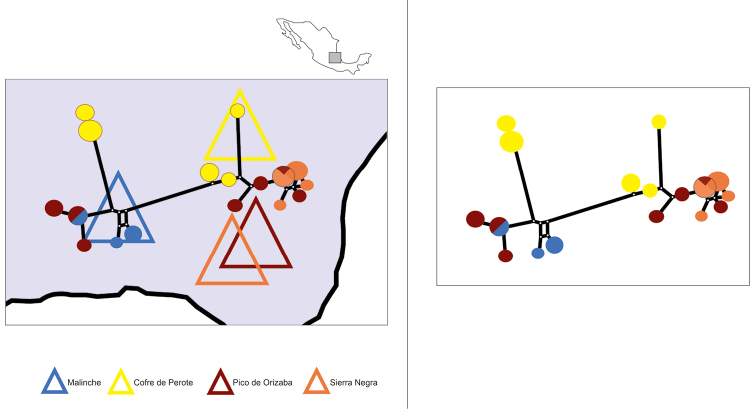
The size of the circles is proportional to the haplotype frequency. The length of links between haplotypes is proportional to the number of mutated positions. The median vectors that represent hypothetical intermediates or unsampled haplotypes are shown in small open dots. The haplotype circles’ color corresponds to the sampling origin (one color per mountain).

Subsequently considering the sample as monospecific, so much variation in the Co1 sequence dataset, obtained from the present locally-restricted sample is promising, providing basic tools for further analyses. Besides, several different mitochondrial lineages were found within the beetle sample, with no clear correspondence between mountains and genetic structure (Fig. 5). Although a group of four close haplotypes could appear to be if not mountain specific, more or less place specific (see the right orange and brown clade sampled from the two closest mountains Cofre de Orizaba and Sierra Negra in Fig. 5), all the main clades group together haplotypes from two or three more or less distant mountains. More importantly, some Co1 haplotypes were found to be shared between individuals sampled from different mountains, including a priori non burrow-connected mountains (Malinche and Pico de Orizaba). Such an apparent lack of geographical structure at the Mexican Volcano Belt scale in a so diverse sequence dataset may suggest either that aerial spread commonly occurs or that some cryptic sub-structure exists, though no conclusion may be drawn from a so small sample. These preliminary results provided a first insight of the phylogeographic structure of the *Geomyphilus pierai*/*Geomyphilus barrerai* complex. A further study using at least Co1 as a marker on a much larger sample should help to elucidate dispersal routes and gene flow of these nidicolous rodent-associated beetles.

## Supplementary materials

Supplementary material 1 EMBL accession numbers of beetle DNA sequences Data type: DNA sequences Explanation note: accession numbers (EMBL) of each Co1 and ITS DNA sequence obtained from beetle individuals and integrated into analyses in the present study. This dataset is made available under the Open Database License (http://opendatacommons.org/licenses/odbl/1.0/). The Open Database License (ODbL) is a license agreement intended to allow users to freely share, modify, and use this Dataset while maintaining this same freedom for others, provided that the original source and author(s) are credited.Alfonsina Arriaga-Jiménez, Lise Roy

## References

[B1] Demastes JW Spradling TA Hafner MS Hafner DJ Reed DL (2002) Systematics and Phylogeography of Pocket Gophers in the Genera Cratogeomys and Pappogeomys. Molecular Phylogenetics and Evolution 22(1): 144–154. doi: 10.1006/mpev.2001.1044 11796037 10.1006/mpev.2001.1044

[B2] Dellacasa M (1987) Contribution to a world-wide catalogue of Aegialiidae, Aphodiidae, Aulonocnemidae, Termitotrogidae (Coleoptera, Scarabaeoidea). Memorie della Societa Entomologica Italiana 66: 1–456.

[B3] Dellacasa M (1988a) Contribution to a world-wide catalogue of Aegialiidae, Aphodiidae, Aulonocnemidae, Termitotrogidae (Part II). Memorie della Societa Entomologica Italiana 67: 1–231.

[B4] Dellacasa M (1988b) Contribution to a world-wide catalogue of Aegialiidae, Aphodiidae, Aulonocnemidae, Termitotrogidae. Addenda et corrigenda. First note. Memorie della Societa Entomologica Italiana 67: 291–316.

[B5] Dellacasa M (1991) Contribution to a world-wide catalogue of Aegialiidae, Aphodiidae, Aulonocnemidae, Termitotrogidae (Coleoptera, Scarabaeoidea). Addenda et corrigenda (second note). Memorie della Societa Entomologica Italiana 70: 3–57.

[B6] Dellacasa M (1995) Contribution to a world-wide catalogue of Aegialiidae, Aphodiidae, Aulonocnemidae, Termitotrogidae (Coleoptera Scarabaeoidea). Addenda et Corrigenda (third note). Memorie della Societa Entomologica Italiana 74: 159–232.

[B7] Dellacasa G Bordat P Dellacasa M (2001) A revisional essay of world genus-group taxa of Aphodiinae (Coleoptera Aphodiidae). Memorie della Societa Entomologica Italiana 79: 1–482.

[B8] Deloya C Lobo JM (1995) Descripción de dos nuevas especies mexicanas de Aphodius de los subgéneros Platyderides y Trichonotulus (Coleoptera: Scarabaeidae: Aphodiinae) asociados con Pappogeomys perriami (Rodentia: Geomyidae). Folia Entomológica Mexicana 94: 41–55.

[B9] De Rojas M Mora MD Ubeda JM Cutillas C Navajas M Guevara DC (2002) Phylogenetic Relationships in Rhinonyssid mites (Acari: Mesostigmata) Based on ribosomal DNA sequences: insights for the discrimination of closely related species. Parasitology Research 88: 675–681. doi: 10.1007/s00436-002-0647-y 12107461 10.1007/s00436-002-0647-y

[B10] Donelly RK Harper GL Morgan AJ Orozco-Terwengel P Pinto-Juma GA Bruford MW (2013) Nuclear DNA recapitulates the cryptic mitochondrial lineages of Lumbricus rubellus and suggests the existence of cryptic species in an ecotoxological soil sentinel. Biological Journal of the Linnean Society 110: 780–795. doi: 10.1111/bij.12171

[B11] Edgar RC (2004) MUSCLE: multiple sequence alignment with high accuracy and high throughput. Nucleic Acids Research 32(5): 1792–1797. doi: 10.1093/nar/gkh340 15034147 10.1093/nar/gkh340 PMC390337

[B12] Fernández JA Hafner MS Hafner DJ Cervantes FA (2014) Conservation status of rodents of the families Geomyidae and Heteromyidae of Mexico. Revista Mexicana de Biodiversidad 85(2): 576–588. doi: 10.7550/rmb.36710

[B13] Folmer O Black M Hoeh W Lutz R Vrijenhoek R (1994) DNA primers for amplification of mitochondrial cytochrome c oxidase subunit I from diverse metazoan invertebrates. Molecular Marine Biology and Biotechnology 3: 294–299. 7881515

[B14] Galtier N Gouy M Gautier C (1996) SEAVIEW and PHYLO_WIN: two graphic tools for sequence alignment and molecular phylogeny. Computer Applications in the Biosciences 12: 543–548. doi: 10.1093/bioinformatics/12.6.543 9021275 10.1093/bioinformatics/12.6.543

[B15] Gordon RD Skelley PE (2007) A monograph of the Aphodiini inhabiting the United States and Canada (Coleoptera: Scarabaeidae: Aphodiinae). Memoirs of the American Entomological Institute 79: 580 pp.

[B16] Guindon S Gascuel O (2003) A simple, fast, and accurate algorithm to estimate large phylogenies by maximum likelihood. Systematic Biology 52(5): 696–704. doi: 10.1080/10635150390235520 14530136 10.1080/10635150390235520

[B17] Hafner MS Light JE Hafner DJ Brant SV Spradling TA Demastes JW (2005) Cryptic species in the Mexican pocket gopher Cratogeomys merriami. Journal of Mammalogy 86: 1095–1108. doi: 10.1644/05-MAMM-A-064R1.1

[B18] Hanski I (1991) North temperate dung beetles. In: Hanski I Cambefort Y (Eds) Dung beetle ecology. Princeton University Press, Princeton, NJ, USA, 75–96.

[B19] Hebert PDN Penton EH Burns JM Janzen DH Hallwachs W (2004) Ten species in one: DNA barcoding reveals cryptic species in the neotropical skipper butterfly Astraptes fulgerator. Proceedings of the National Academy of Sciences of the United States of America 101: 12–17. doi: 10.1073/pnas.0406166101 10.1073/pnas.0406166101 PMC522015 15465915

[B20] Librado P Rozas J (2009) DnaSP v5: A software for comprehensive analysis of DNA polymorphism data. Bioinformatics 25: 1451–1452. doi: 10.1093/bioinformatics/btp187 19346325 10.1093/bioinformatics/btp187

[B21] Lobo JM Halffter G (1994) Relaciones entre escarabajos (Coleoptera: Scarabaeoidea) y nidos de tuza (Rodentia: Geomyidae): Implicaciones biologicas y biogeográficas. Acta Zoológica Mexicana 62: 1–9.

[B22] Navajas M Lagnel J Gutierrez J Boursot P (1998) Species-wide homogeneity of nuclear ribosomal ITS2 sequences in the spider mite Tetranychus urticae contrasts with extensive mitochondrial COI polymorphism. Heredity 80: 742–752. doi: 10.1046/j.1365-2540.1998.00349.x 9675873 10.1046/j.1365-2540.1998.00349.x

[B23] Rozas J Rozas R (1995) DnaSP, DNA sequence polymorphism: an interactive program for estimating Population Genetics parameters from DNA sequence data. Computer Applications in the Biosciences 11: 621–625. doi: 10.1093/bioinformatics/11.6.621 8808578 10.1093/bioinformatics/11.6.621

[B24] Skelley PE Dellacasa M Dellacasa G Gordon RD (2007) Checklist of the Aphodiini of Mexico, Central and South America (Coleoptera: Scarabaeidae: Aphodiinae). Insecta Mundi 0014: 1–14.

[B25] Wirta H (2009) Complex phylogeographical patterns, introgression and cryptic species in a lineage of Malagasy dung beetles (Coleoptera: Scarabaeidae). Biological Journal of the Linnean Society 96(4): 942–955. doi: 10.1111/j.1095-8312.2008.01156.x

