# Supplementary material for: Co1 DNA supports conspecificity of Geomyphilus pierai and G. barrerai (Coleoptera, Scarabaeidae, Aphodiinae) and is a good marker for their phylogeographic investigation in Mexican mountains
Source: Zookeys. 2015 Jul 6;(512):77–88. doi: 10.3897/zookeys.512.9646 (PMC4523755; doi:10.3897/zookeys.512.9646)
Supplement: Supplementary material 1 — EMBL accession numbers of beetle DNA sequences [file zookeys-512-077-s001.docx]

**Appendix (Online supplementary materials)**

EMBL accession numbers* of beetle DNA sequences

| No. Ind | Species | Individual ID | Accession # | |
| --- | --- | --- | --- | --- |
|  |  |  | Co1 | ITS |
| 1 | *G. pierai* | V8R_Gpierai | LN832250 | LN832245 |
| 2 | *G. pierai* | P2r_Gpierai | LN832251 | LN832197 |
| 3 | *G. barrerai* | M2r_Gbarrerai | LN832252 | LN832214 |
| 4 | *G. barrerai* | S1r_Gbarrerai | LN832253 | NA |
| 5 | *G. barrerai* | S3r_Gbarrerai | LN832254 | NA |
| 6 | *G. pierai* | S3r_Gpierai | NA | LN832208 |
| 7 | *G. barrerai* | S5r_Gbarrerai | LN832255 | NA |
| 8 | *G. pierai* | S5r_Gpierai | NA | LN832204 ; LN832205 ^+^ |
| 9 | *G. pierai* | P1r_Gpierai | LN832256 | LN832198 ; LN832199 ^+^ |
| 10 | *G. barrerai* | M1r_Gbarrerai | LN832257 | LN832213 |
| 11 | *G. barrerai* | P1r_Gbarrerai | LN832258 | LN832219 |
| 12 | *G. barrerai* | P3r_Gbarrerai | LN832259 | LN832217 |
| 13 | *G. barrerai* | P4r_Gbarrerai | LN832260 | LN832216 |
| 14 | *G. pierai* | M1r_Gpierai | LN832261 | NA |
| 15 | *G. pierai* | M2r_Gpierai | LN832262 | NA |
| 16 | *G. pierai* | P3r_Gpierai | LN832263 | LN832196 |
| 17 | *G. pierai* | V1R_Gpierai | LN832264 | LN832195 |
| 18 | *G. pierai* | V2R_Gpierai | LN832265 | LN832194 |
| 19 | *G. pierai* | V3R_Gpierai | LN832266 | LN832192 |
| 20 | *G. pierai* | V4R_Gpierai | LN832267 | LN832193 |
| 21 | *G. pierai* | V5R_Gpierai | LN832268 | LN832191 |
| 22 | *G. pierai* | P12r_Gpierai | LN832269 | LN832232; LN832233 ^+^ |
| 23 | *G. pierai* | P14r_Gpierai | LN832270 | LN832236 |
| 24 | *G. pierai* | S6r_Gpierai | LN832271 | LN832239 |
| 25 | *G. pierai* | S9r_Gpierai | LN832272 | LN832242 |
| 26 | *G. pierai* | V7R_Gpierai | LN832273 | LN832244 |
| 27 | *N. perotensis* | V9R_Nperotensis | LN832274 | LN832247 |
| 28 | *N. perotensis* | V10R_Nperotensis | LN832275 | LN832248 |
| 29 | *N. perotensis* | V6R_Nperotensis | LN832276 | LN832243 |
| 30 | *G. pierai* | S8r-Gpierai | LN832277 | LN832241 |
| 31 | *G. pierai* | S4r-Gpierai | LN832278 | LN832206; LN832207 ^+^ |
| 32 | *G. pierai* | V12R_Gpierai | LN832279 | LN832246 |
| 33 | *G. pierai* | C9r_Gpierai | LN832280 | LN832227 |
| 34 | *O. vacca* | Ontho_ma | LN832282 | LN832190 |
| 35 | *O. similis* | Ontho_sim | LN832281 | LN832189 |
| 36 | *G. pierai* | C5R_Gpierai | NA | LN832200 |
| 37 | *G. pierai* | C2R_Gpierai | NA | LN832201 |
| 38 | *G. pierai* | C1R_Gpierai | NA | LN832202 ; LN832203^+^ |
| 39 | *G. pierai* | S2r_Gpierai | NA | LN832209 ;LN832210^+^ |
| 40 | *G. barrerai* | P5r_Gbarrerai | NA | LN832215 |
| 41 | *G. barrerai* | P2r_Gbarrerai | NA | LN832218 |
| 42 | *G. pierai* | CR_Gpierai | NA | LN832220 |
| 43 | *G. barrerai* | Mr_Gbarrerai | NA | LN832221 |
| 44 | *G. pierai* | M8r_Gpierai | NA | LN832222 |
| 45 | *G. pierai* | Pr_Gpierai | NA | LN832223 |
| 46 | *G. pierai* | C6R_Gpierai | NA | LN832224 |
| 47 | *G. pierai* | C7R_Gpierai | NA | LN832225 |
| 48 | *G. pierai* | C8R_Gpierai | NA | LN832226 |
| 49 | *G. pierai* | C10r_G.pierai | NA | LN832228 |
| 50 | *G. pierai* | C11r_Gpierai | NA | LN832229 |
| 51 | *G. pierai* | C12r_Gpierai | NA | LN832230 |
| 52 | *G. pierai* | P11r_Gpierai | NA | LN832231 |
| 53 | *G. pierai* | P13r_Gpierai | NA | LN832234 ; LN832235 ^+^ |
| 54 | *G. pierai* | P15r_Gpierai | NA | LN832237 ; LN832238 ^+^ |
| 55 | *G. pierai* | S7r_Gpierai | NA | LN832240 |
| 56 | *N. perotensis* | V11R_Nperotensis | NA | LN832249 |
| 57 | *G. pierai* | S1r_Gpierai | NA | LN832211 ; LN832212^+^ |

* DNA sequences are currently under submission for EMBL accession numbers assignation.

^+^ sequences of allele 1 and allele 2
